# Mumio and bladder cancer: unlocking its potential in 3D cell culture

**DOI:** 10.1186/s12885-026-15838-1

**Published:** 2026-03-14

**Authors:** Zuzanna Fekner, Julia Sieracka, Paweł Dąbrowski, Łukasz Kaźmierski, Marta Rasmus, Arkadiusz Jundziłł, Tomasz Drewa, Dariusz Grzanka, Marta Pokrywczyńska, Tomasz Kloskowski

**Affiliations:** 1 Department of Regenerative Medicine, Chair of Urology and Andrology, Collegium Medicum, Nicolaus Copernicus University, Bydgoszcz, Poland; 2 Institute of Advanced Studies, IAS, Nicolaus Copernicus University, Toruń, Poland; 3 Department of Clinical Pathomorphology, Collegium Medicum, Nicolaus Copernicus University, Bydgoszcz, Poland

**Keywords:** 3D cell culture, Mumio, Bladder cancer, Spheroids, Cytotoxicity, Cell cycle arrest

## Abstract

**Background:**

Bladder cancer remains one of the most prevalent causes of cancer-related mortality worldwide. Mumio, a naturally occurring substance with a long-standing history of use in traditional medicine, has demonstrated therapeutic potential, including anticancer properties. This study aimed to evaluate the effects of a pharmaceutical formulation of Mumio on the growth of normal (SV-HUC-1) and cancerous (T24, 5637) bladder cell lines in 3D culture.

**Methods:**

Spheroids were generated using ultra-low attachment 96-well plates. Three concentrations of Mumio (LC₁₀, LC₅₀, LC₉₀), previously determined in 2D culture, were applied. Cells were incubated for 24, 48, and 72 h. Cell viability and caspase activity in 3D culture were assessed via luminescence-based assays. Hematoxylin–eosin (HE) and Ki-67 staining were performed to evaluate spheroid morphology and proliferation. Gene expression analysis was conducted to investigate molecular mechanisms.

**Results:**

Mumio exhibited dose-dependent cytotoxicity in 3D culture, with T24 cells showing the highest sensitivity compared to the control and the second bladder cancer cell line (5637) after all incubation times. Concentrations effective in 2D culture were less potent in 3D conditions. Proliferation rates decreased with increasing Mumio concentration and prolonged exposure. Molecular analysis revealed G1 phase cell cycle arrest and reduced DNA synthesis.

**Conclusions:**

Mumio has potential in supportive bladder cancer treatment; however, its effectiveness, as observed in 2D culture, exhibited diminished efficacy when applied to 3D cultures. Further investigations are necessary, including the use of organoids or animal models, to confirm these properties.

**Supplementary Information:**

The online version contains supplementary material available at 10.1186/s12885-026-15838-1.

## Background

Mumio is a natural substance whose history of use in traditional folk medicine dates back to antiquity. Mumio is a herbal-mineral exudate that ranges in color from pale brown to black-brown. Over the years, numerous hypotheses have been proposed regarding its origin, but most assume that the formation of Mumio is a long-term process, during which mineral rocks and plant remains play important roles [1–6]. This substance is found in caves, crevices, rock depressions, and cliffs at altitudes ranging from 1,000 to 5,000 m above sea level in many mountain ranges worldwide, particularly in the Himalayan ranges and mountainous regions of India [1, 2, 4–6]. Difficult conditions of occurrence make Mumio not an easy substance to extract. It is most often found in the form of dripstones, and in dry places sheltered from wind and sun, it can also be found in the form of salt deposits [1, 4, 7, 8].

Mumio is well known in various parts of the world, but it is most common in Asian countries. This substance is known by various names, depending on its location. The most commonly used ones include: Shilajit, Saljit, Gujarati (in the regions of India), Shilajatu (in Bengali), Moomiaii, Munmnaei (in the region of Persia), Mumijo, Mymiyo (in Russia), Mumie (in German), Kao-Tun (in Burma), and many others [1, 5]. The word Shilajit consists of two parts: "Shila" which means rock and "jit" which means victory, so its literary meaning can be described as "conqueror of mountains". On the other hand, the Sanskrit word Shilajit is translated as "conqueror of mountains and destroyer of weakness" [6]. Mumio also has many colloquial names referring to the method of its formation, extraction, appearance, or consistency, such as "mountain juice", "rock juice", "mountain sweat", "mountain blood", "mineral tar", "asphalt", and "mountain oil" [3, 5–7, 9].

Despite its occurrence in many places on Earth, the qualitative chemical composition of Mumio is very similar. However, samples from individual regions differ significantly in the percentage of some components [1, 6, 10]. The chemical composition of Mumio is influenced by numerous factors, including geographical and environmental factors, as well as the presence of minerals, native plants, molds, and bacteria, altitude, temperature, atmospheric pressure, and geothermal pressure [2, 6, 10]. The chemical composition of Mumio mainly includes organic matter (60–80%), inorganic matter (20–40%), and trace amounts of elements (the most common are: potassium, calcium, magnesium, and iron) [6, 9]. The predominant part of organic compounds are humic substances, which are the product of biochemical transformations of dead plants, microbial remains, and fauna, as a result of the action of many microorganisms. Humic substances are divided into three main fractions: humins, humic acids, and fulvic acids [11]. The composition of Mumio is responsible for its antioxidant and antimicrobial properties [12]. This substance is also known for its osteogenic properties [9, 13].

**Fig. 1:**
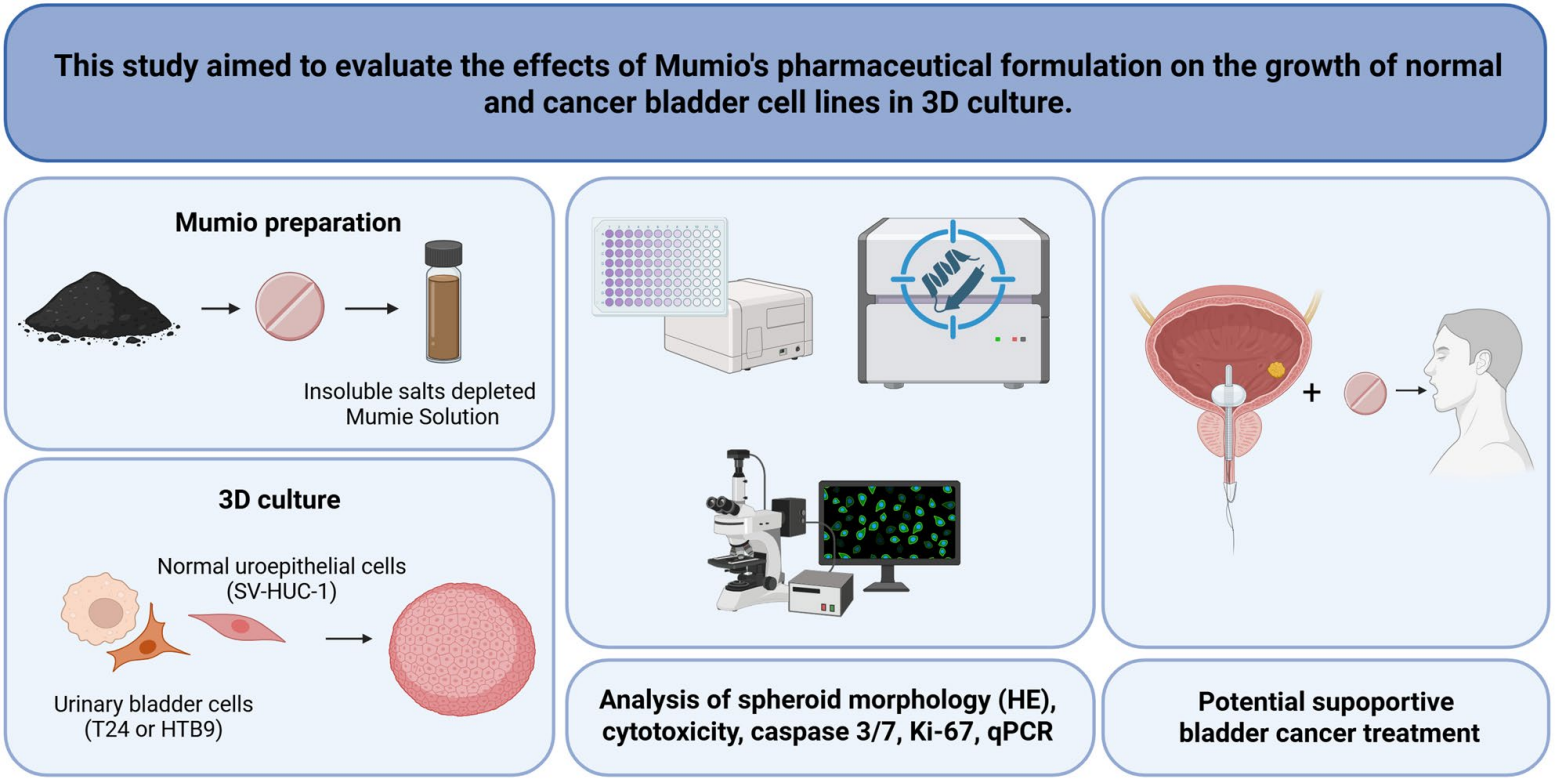
Study design. Schematic representation of the study protocol and potential application of Mumio in clinical practice

Due to its wide range of applications in traditional medicine, Mumio is the subject of many studies aimed at confirming its valuable properties and therapeutic potential [1]. In recent years, there has been an increased interest in this substance as a potential anticancer compound. In a previous study, we conducted an experiment on Mumio in 2D culture, demonstrating that this substance exhibits promising properties for supporting bladder cancer treatment. To confirm these properties, this study continues the research using 3D culture (Fig. 1). Cell culture in the form of spheroids better reflects in vivo conditions.

## Methods

### Cell lines

The cell lines used in the study were obtained from the American Type Culture Collection (ATCC, USA). A normal urothelial cell line (SV-HUC-1) was used as a control. The study groups were the following cell lines: human transitional cell carcinoma of the urinary bladder (T24) and human stage II bladder carcinoma (5637). SV-HUC-1 cells were cultured in F-12 K medium with L-glutamine (Corning, USA) supplemented with 10% inactivated fetal bovine serum (BIOWEST, Sigma Aldrich, USA) and antibiotics: 5 μg/ml amphotericin B (Corning, USA) and 100 μg/ml streptomycin with 100 U/ml penicillin (Corning, USA). T24 cells were cultured in DMEM/Ham’s F-12 50/50 medium, while 5637 cells were cultured in RPMI-1640 medium. Both media were supplemented with L-glutamine and HEPES (Corning, USA), using the same supplements as those used for SV-HUC-1 cells. Cells prior to passage 10 were used in the study.

### Mumio extracts

To prepare Mumio extracts, commercially available tablets containing 200 mg of Mumio (OOO FARMGRUP, Russia) were used. The solution was prepared by dissolving the tablet in 10 mL of culture medium and then centrifuging for 10 min at 500 × g to precipitate insoluble organic components. The supernatant was filtered through a 0.22 μm syringe filter (Merck Millipore, USA) to obtain a pure stock solution at a concentration of 20 mg/ml. The prepared solution was used to obtain the appropriate lethal concentrations (LC) for each time interval and cell type, calculated in a previous study [1].

### 3D cell culture

To obtain spheroids, cells from each tested cell line were seeded onto 96-well ultra-low attachment round-bottom plates (PrimeSurface 96U 3-D cell culture plate, S-BIO, Japan) at a density of 25,000 cells per well. The seeded plates were centrifuged at 200 × g for 10 min and then placed in an incubator for culture for 3 to 4 days. After this time, previously prepared solutions containing appropriate concentrations of Mumio were added to the spheroids and incubated for 24, 48, and 72 h.

### Luminescence assays

The CellTiter-Glo® 3D assay (Promega, Germany) was used to measure spheroid viability. The day before analysis, the reagent was thawed at 4 °C and heated for 30 min at 22 °C immediately before use. Spheroids suspended in 50 μl of culture medium were transferred to a 96-well white plate (FALCON, USA), and next an equal volume of the reagent was added. The plate was placed in a Varioskan LUX plate reader (Thermo Fisher Scientific, USA), shaken for 5 min at 420 rpm, and incubated in the dark for 30 min. Afterward, the luminescence signal was measured. The assay was repeated three times for each cell line and each incubation time with Mumio.

The Caspase-Glo®3/7 3D assay (Promega, Germany) was used to measure caspase activity. The assay reagent was prepared by combining the substrate with buffer and then placing it in a refrigerator (4 °C). Before starting the experiment, the prepared reagent was left at room temperature. As in the viability assay, the spheroids were transferred to a new white 96-well plate, an equal volume of reagent was added, and then the plate was placed in the plate reader. The caspase activity assay protocol was similar to the viability assay, except for the shaking parameters (30 s at 500 rpm).

### Morphological and morphometric evaluation

A Leica DMi1 inverted light microscope (Leica, Germany) was used to create photographic documentation of spheroids after Mumio incubation. Morphometric analysis, which involved measuring the surface area of spheroids, was performed using EPview 1.4 software (Olympus, Japan).

### *S-LC*_*50*_* calculation*

Based on the viability results of spheroids incubated with three different concentrations calculated earlier (in the 2D study), the LC_50_ for cells in 3D culture was calculated (S-LC_50_). Calculation was performed by fitting a trend line to the obtained viability results and then calculating the S-LC_50_ values ​​from the obtained formulas.

### Histochemical and immunohistochemical evaluation

To fix the spheroids, they were transferred to 1.5 ml tubes (Eppendorf, Germany), washed twice in 200 μl PBS, then 100 μl of 4% paraformaldehyde (Polysciences, USA) was added for 10 min of incubation at room temperature. After this time, the paraformaldehyde was removed, and the spheroids were suspended in 200 μl PBS until further analysis. The fixed spheroids were used to prepare slides. In the first stage, the spheroids were suspended in a small amount (30–50 μl) of histo-gel (Erpedia, Germany). After solidification of the gel with the spheroids, it was placed in 50% ethanol. The samples prepared in this manner were sent to the Department of Clinical Pathomorphology at the Collegium Medicum, Nicolaus Copernicus University. To prepare the slides, the samples were embedded in paraffin, sectioned, and then stained. Histochemical staining with hematoxylin and eosin (HE) was used to visualize cell nuclei and cytoplasm. Proliferative activity was assessed by immunohistochemistry using the Ki-67 antibody. Staining was performed according to the manufacturer's protocol. Photographic documentation was performed using an EP50 (Olympus, Japan).

### Quantitative, image-based proliferation

Analysis of spheroids proliferation was performed based on slides stained with the Ki-67 antibody. Images were acquired using an EP50 (Olympus, Japan) color sensor sCMOS camera mounted on an inverted CX53 microscope. Images were then post-processed, including background subtraction and shading correction, and analyzed via CellSen Dimensions 4.2 software with the Count and Measure add-on. After determining that the HSV thresholding method is well-suited for analyzing Ki-67-positive-stained cells, we performed the detection and segmentation of these objects. If any objects outside the spheroid or debris were found to be Ki-67 positive, they were manually removed. Additionally, if multiple spheroids were present in a single frame, we analyzed them separately. Next, we determined the spheroid area using manual ROI (region of interest) segmentation. Data regarding the total spheroid area and Ki-67 positive cells within this area are presented as a ratio (Ki-67/total spheroid area).

### Molecular analysis

Spheroids treated with Mumio extracts were analyzed by real-time PCR to assess expression of selected genes. Total RNA was extracted using the RNeasy Mini Kit (Qiagen, Germany) and quantified with a NanoDrop Lite spectrophotometer (Thermo Fisher Scientific, USA). cDNA was synthesized using the RT^2^ First Strand Kit (Qiagen, Germany).

Gene expression was determined with the RT^2^ SYBR Green qPCR Mastermix and a Custom RT^2^ PCR Array (Qiagen, Germany) containing primers for TOP1, TOP2A, TOP2B, TOP3A, TOP3B, CASP3, CASP7, CASP9, BAX, BCL2, CDK1, CDK2, CDK4, CDK6, CDK7, CDKN1A, CDKN1B, and TP53, as well as reference genes (B2M, HPRT1, RPLP0), genomic DNA control (HGDC), reverse transcription control (RTC), and positive PCR control (PPC) (Supp. Figure 1). Reactions were performed on a LightCycler 480 II (Roche, Switzerland) following the RT^2^ Profiler PCR Array Handbook (Qiagen). Reference genes were selected using NormFinder, and relative quantification was applied to determine expression changes. Statistical analysis was performed on all data. Genes were considered significantly regulated when p < 0.05. Given the targeted nature of the gene panel, fold change (FC) values were reported as effect size rather than used as a filtering criterion for differential expression. For descriptive purposes, expression changes were further categorized as strong (FC ≥ 1.5 or ≤ 0.67), moderate (FC 1.3–1.5 or 0.77–0.67), or weak (FC < 1.3 and > 0.77).

### Statistical analysis

Statistical analysis was performed using GraphPad Prism (GraphPad Software 8.4., USA). Data were calculated based on the average of at least three replicates for each experiment. Results were presented as means ± standard deviation (SD). The Shapiro–Wilk test was used to assess the normality of the distribution. Statistical analysis was performed using one-way ANOVA (for cell viability) or two-way ANOVA (for group analysis). For results with a non-normal distribution, the Kruskal–Wallis and Dunn's multiple comparison tests were used. Graphs illustrating statistical data were generated using Graphpad Prism.

## Results

### Influence of Mumio on morphological and morphometric spheroid parameters

**Fig. 2:**
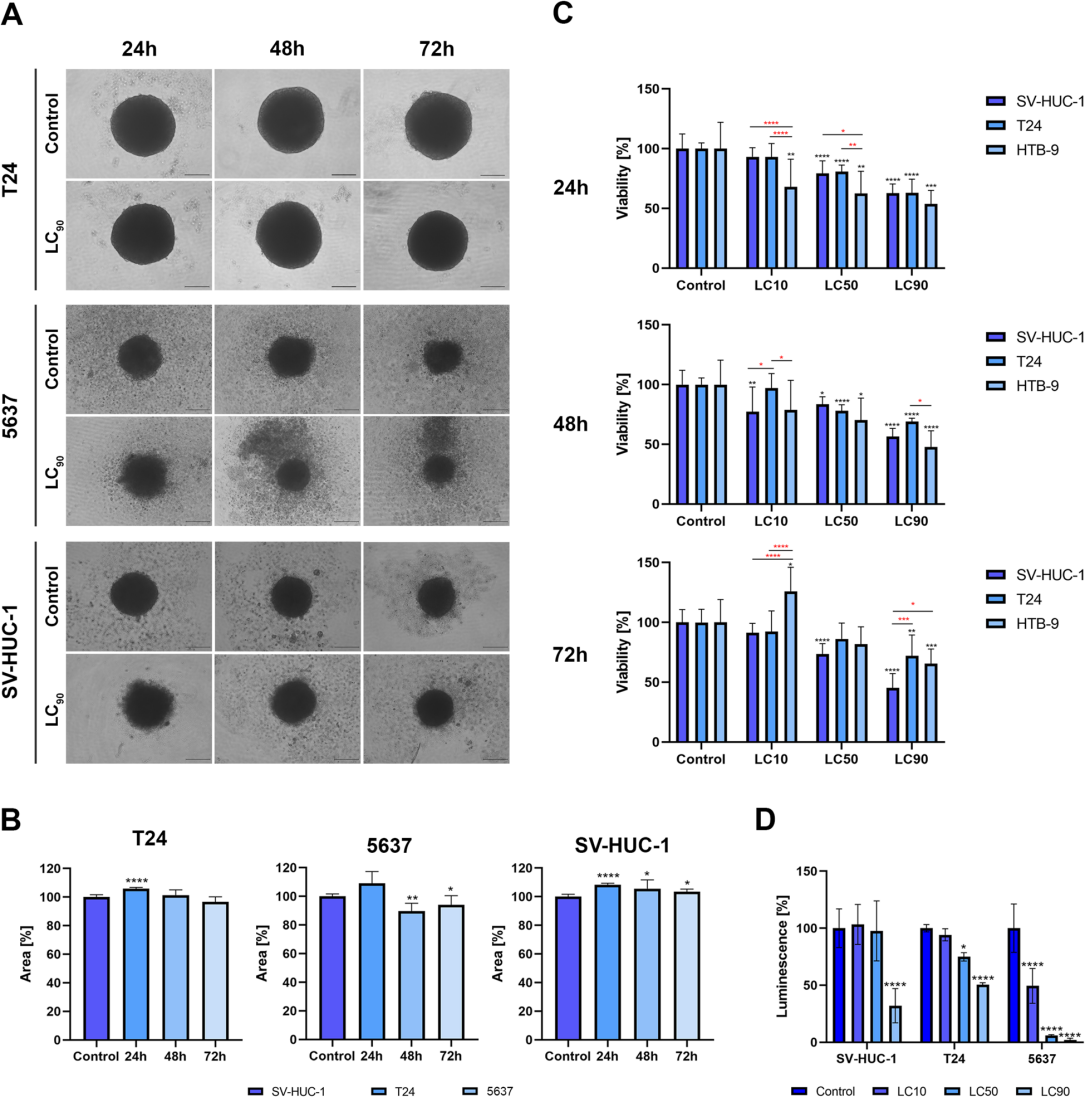
Cytotoxic effect of Mumio on tested spheroids. **A** Lack of significant changes in spheroid morphology compared to the control was observed. Inverted light microscope, scale bar = 200 µm. **B** Slight changes in the spheroids' surface after incubation with Mumio were observed. Results were presented for the control and the LC90 group. **C** Mumio reduced spheroids' viability in a concentration-dependent manner. **D** Reduction in Caspase 3/7 activity was observed after incubation with Mumio extract. The values are presented as means ± SD. *p ≤ 0.05, **p ≤ 0.01, ***p ≤ 0.001, ****p ≤ 0.0001. The black asterisks indicate differences relative to the control, and the red asterisks indicate differences between the tested groups. Data are from four biological replicates (*n* = 4)

Morphological analysis in most cases showed a slight change in spheroid diameter with increasing Mumio concentration (Fig. 2A,B; Supp. Figure 2 A,B). In the case of the SV-HUC-1 cell line, a statistically significant, although modest, increase in spheroid surface area was observed at the LC_90_ concentration after each incubation time. Application of the LC_10_ concentration did not result in significant changes at any time interval, except for 72 h of incubation, where each concentration had a statistically important effect on spheroid size (Fig. 2A,B; Supp. Figure 2 A,B). In the T24 cell line, a statistically important increase in spheroid diameter was observed at each concentration after 24 h of incubation. Longer incubation time did not result in significant changes in spheroid size (Fig. 2A,B; Supp. Figure 2 A,B). The greatest diversity in spheroid size was observed in the case of the 5637 cell line, where, after 48 and 72 h of incubation at the LC_90_ concentration, there was a statistically significant decrease in spheroid surface area. In the remaining cases, the spheroid area increased slightly (LC_10_ and LC_50_ after 72 h of incubation), or no statistically important difference in size was observed compared to the control (Fig. 2A, B; Supp. Figure 2B).

### Spheroids' viability and caspase activity

Mumio has been shown to reduce cell viability in a dose-dependent manner (Fig. 2C). The cytotoxic effect of Mumio in 3D culture was less effective compared to standard 2D culture [1]. Calculated LC values did not reduce cell viability by 10%, 50%, or 90%. The lowest detected viability was at 50% in the highest tested concentration. No significant differences in viability were observed between the tested cells. Only 5637 spheroids, after 24-h incubation in LC_10_ and LC_50_, were more sensitive to Mumio action compared to control SV-HUC-1 spheroids. In most cases, no difference in viability was observed between spheroids incubated for different times (Supp. Figure 2 C).

The assessment of caspase activity using the Caspase-Glo®3/7 3D assay showed a decrease in caspase activity with increasing Mumio concentration in all cell lines. The most significant decrease was observed in the LC_50_ and LC_90_ concentrations for the 5637 cell line (Fig. 2D).

### Mumio effect on spheroids

Based on viability results, we calculated LC_50_ values for cells cultured in 3D culture (S-LC_50_, Table 1). The results showed that the most sensitive cell line after all incubation times with Mumio was T24. In all cases, the SI value was greater than 1, indicating selectivity against the T24 cancer cell line. The second tested cancer cell line (5637) was more sensitive to Mumio action compared to the control after 24 h of incubation. This difference decreased with increasing incubation time, as evidenced by a decrease in the selectivity index (Table 1).

**Table 1:** LC_50_ values calculated for spheroids (S-LC_50_) treated with different Mumio concentrations. Data are from four biological replicates, *n* = 4

S-LC_50_ [µg/mL]	24h	48h	72h
SV	9268.7	5983.5	3260.8
T24	6527.1	3257.9	2177.5
SI_T24_	*1.42*	*1.84*	*1.50*
5637	6695.6	5751.1	3870.4
SI_5637_	*1.38*	*1.04*	*0.84*

*S-LC* Spheroid-Lethal Concentration, *SI* Selectivity Index

### Evaluation of proliferation and cell morphology inside spheroids

**Fig. 3:**
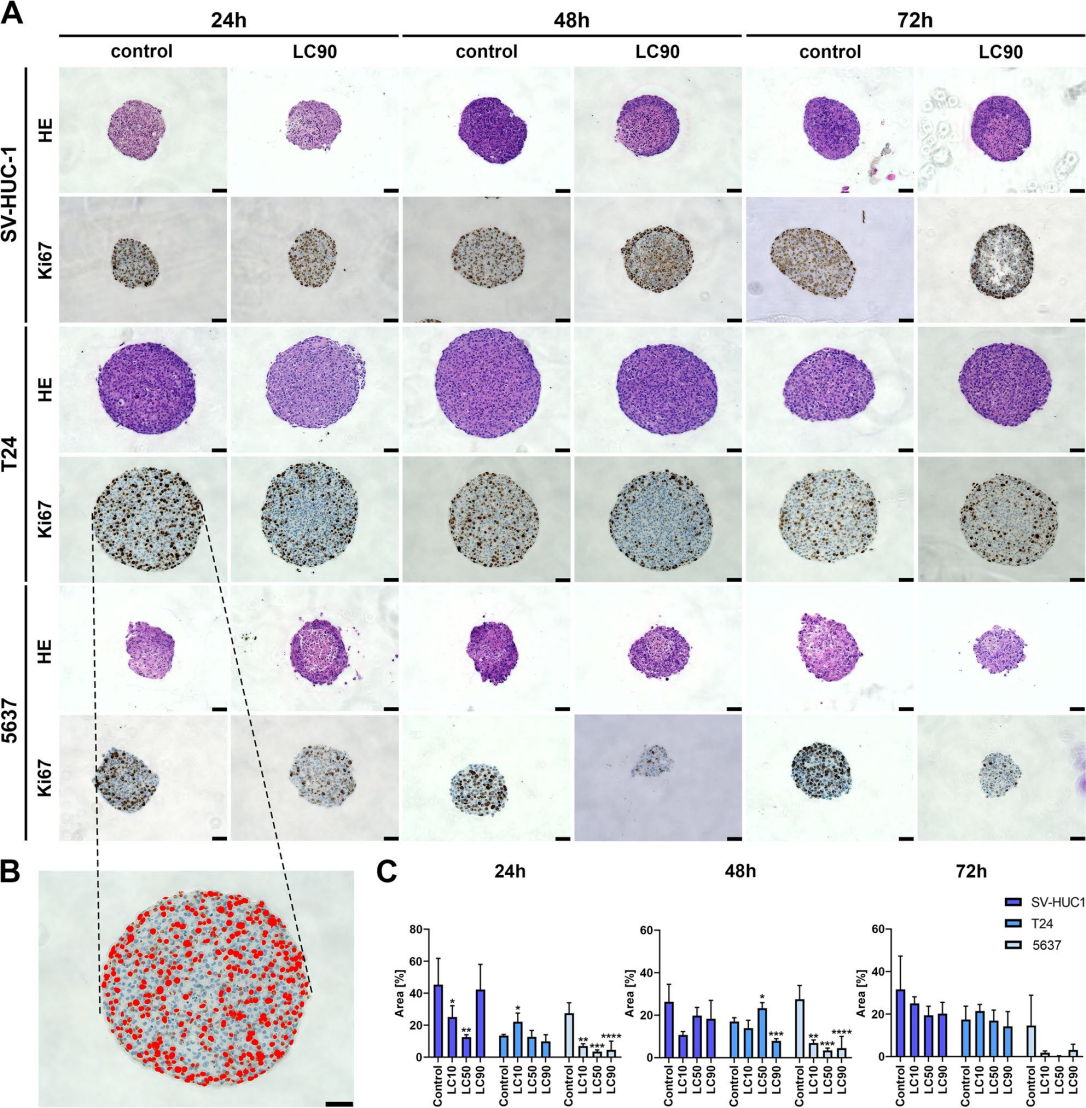
Histological and immunohistochemical evaluation. **A** Haemoxylin and eosin staining showed a lack of significant changes in the morphology of cells creating spheroids after Mumio treatment; in some cases, the brightly coloured cytoplasm of the cells is visible in the spheroid central part. Expression of the Ki67 marker decreased in the highest Mumio concentration, especially in the central area of the spheroids. **B** Example of HSV thresholding of tested slides for numerical analysis of Ki67 expression. **C** Quantitative Ki67 expression showed a reduction in the number of cells actively proliferating after incubation with a higher Mumio concentration. The values are presented as means ± SD. *p ≤ 0.05, **p ≤ 0.01, ***p ≤ 0.001, ****p ≤ 0.0001. Data are from three biological replicates (*n* = 3)

HE staining showed the presence of regular nuclei in spheroids after 24, 48, and 72 h of incubation with Mumio in all cell lines (Fig. 3A, Supp. Figure 3). There were slight differences in the intensity of staining of nuclei and cytoplasm between the individual incubation times with Mumio in the SV-HUC-1 and 5637 groups, especially after incubation in the highest tested concentration. Spheroids formed from the T24 cancer line were significantly larger than spheroids from the other cell lines. Similar to normal cells, they primarily assumed a round, compact shape with evenly stained and distributed cell nuclei (Fig. 3A, Supp. Figure 3).

Ki-67 staining showed a strong intensity, mainly in the outer layers of the spheroids. In their central parts, the Ki-67 signal was weaker. The Ki-67 signal was evenly distributed in the cell nuclei. A relationship was observed between the incubation time and the concentration used, as well as the intensity of Ki-67 staining. With increasing Mumio concentration and extending the incubation time, the number of stained proteins decreased (Fig. 3A, Supp. Figure 3).

### Numerical representation of KI67 expression

The results show a clear dose- and time-dependent effect of the Mumio on the proliferation ratio of different cell lines. In most spheroids tested, an increase in the drug dose resulted in a decrease in cell proliferation rates, as indicated by a drop in Ki-67-positive cells relative to the total area of the spheroid. The proliferation rate of 5637 cells decreased the most among the three cell lines at all incubation times and in response to all concentrations tested. After 72 h, the drop in Ki-67 positive cells per spheroid area for this cell line was 12.71% when comparing the LC_10_ group to the control (Fig. 3C). It is worth noting that as spheroid growth progresses, the number of Ki-67 positive cells might drop due to the rising demand for nutrients and the growing hypoxic nucleus of the spheroid. Consequently, we might observe a decrease in cell proliferation rates in the overgrown control spheroids after prolonged culture, as was observed after 48 or 72 h in our experiment.

### Changes in gene expression

**Fig. 4:**
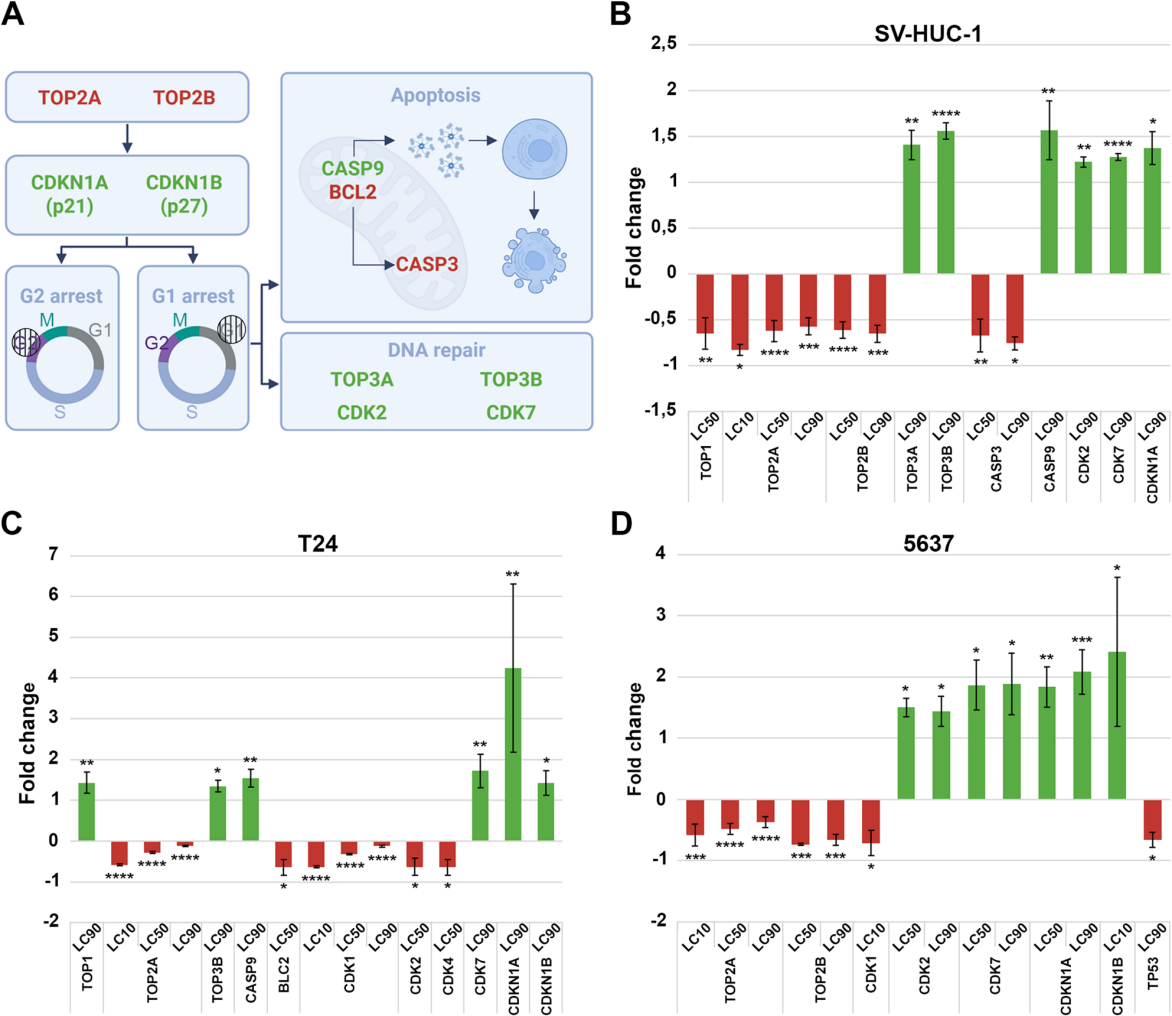
Effect of Mumio on molecular pathways. Proposed mechanism of action of Mumio based on the results obtained from molecular analysis (**A**). Differentially expressed genes in SV-HUC-1 (**B**), T24 (**C**), and 5637 (**D**) spheroids after Mumio treatment. RNAs were collected after 24 h of incubation of cells with Mumio. The values are presented as means ± SD. *p ≤ 0.05, **p ≤ 0.01, ***p ≤ 0.001, ****p ≤ 0.0001. Data are from four biological replicates (*n* = 4)

The analysis of the studied genes was performed against the reference gene HPRT1 for SV-HUC-1 and 5637 cell lines and B2M for the T24 cell line. Molecular analysis revealed a variable gene expression profile in the studied cell lines (Fig. 4). In cancer cell lines, mostly high and some moderate changes in gene expression were observed. In the control SV-HUC-1 cell line, in half of the cases, a strong change in expression was observed; in the remaining cases, the changes were moderate or small (Supp. Tab. 1–3).

In all tested groups, downregulation of TOP2A and TOP2B was observed. Genes involved in the apoptosis process were downregulated in SV-HUC-1 (CASP3) and T24 (BCL2) spheroids. The CDKN1A gene was downregulated in the lowest tested concentration in T24 and 5637 spheroids. In the T24 group, downregulation of CDK1, CDK2, and CDK4 was noticed. Change in TP53 gene expression was detected only in 5637 spheroids (downregulation) (Fig. 4B-D, Supp. Tab.1–3).

In the SV-HUC-1 and T24 groups, gene upregulation was observed only in the highest tested concentration (LC_90_). In both spheroid types, increased expression in TOP3B, CASP9, CDK7, and CDKN1A was observed. Differences were observed in TOP3A and CDK2 for SV-HUC-1, and in TOP1 and CDKN1B for T24. In the case of 5637 spheroids at the lowest concentration (LC_10_), CDKN1B was upregulated, whereas at higher concentrations (LC_50_ and LC_90_), the CDK2, CDK7, and CDKN1A genes were upregulated (Fig. 4B-D, Supp. Tab. 1–3).

## Discussion

Cancers are a serious problem in contemporary medicine. Currently used therapies are often insufficient, which is why scientists are constantly looking for new substances with better anti-cancer potential. Natural compounds and their synthetic derivatives hold great potential in this field due to their high chemical diversity, biochemical specificity, significant molecular activities, and pharmacological properties [14]. Mumio has been used in traditional medicine for thousands of years. In recent years, there has been growing interest in this substance as a potential cancer treatment, as evidenced by the increasing number of publications on this topic.

Currently, Mumio is commonly used as a supplement for various conditions in different forms, including natural (resin) and pharmaceutical (tablet) forms. Despite that, the safety of Mumio has been assessed in several studies. Velmurugan and colleagues examined the effect of repeated administration of Mumio to rats at different doses for 91 days. They demonstrated that long-term consumption is safe and does not cause significant changes in the animals' organs, except for the highest dose (5000 mg/kg), for which minor changes were observed in the liver, correlating with an excess of iron in this organ [15]. Mumio is a rich source of this element [6, 16]. The safety of oral administration of Mumio in humans was examined, among others, by Keller et al. They performed a study on the effect of Mumio supplementation on fatigue-induced decreases in muscle strength and serum hydroxyproline levels. In 2019, they conducted a study on a group of 63 healthy men, which showed the advantage of 8-week supplementation with a high dose of Mumio (500 mg) compared to a low dose (250 mg) and placebo on maintaining muscle strength after heavy exercise. No adverse events were reported during the study, indicating the safety of the administered doses [17]. In 2020, Sadeghi et al. conducted a randomized, double-blind clinical trial to assess the effect of oral intake of 500 mg of Mumio capsules on tibial fracture repair. The study demonstrated that the use of Mumio significantly shortened the tibial bone regeneration period compared to the placebo, without increasing the frequency of adverse events [18]. The results of these studies indicate that oral intake of 500 mg of Mumio for several weeks is safe and does not cause adverse events, suggesting the possibility of testing this dose in other disease entities. Mumio has also been studied for its effectiveness in treating urinary tract diseases and reproductive system dysfunctions [19, 20]. A 1997 study of 38 patients showed that Mumio may have a beneficial effect on bladder obstruction in patients with mild prostatic hyperplasia [20]. Data analysis revealed that in the vast majority of analyzed Mumio samples, levels of heavy metals were below the permissible limits set by the WHO and the FDA. Additionally, the humic substances present in Mumio can chelate toxic heavy metals. The authors of this study indicate that Mumio consumption without knowing the levels of metals is not safe [21].

In our previous study, we evaluated the effect of Mumio on these same three cell lines in 2D culture [1]. Cytotoxicity towards one of the tested cancer cell lines (T24) was higher compared to normal cells of the same organ. In the case of the 5637 cell line, a stronger cytotoxic effect, compared to the control group, was observed only after 72 h of exposure to Mumio. Based on the cytotoxic results, the LC value was calculated, which we used in this study on a 3D culture model to determine whether the results obtained were comparable to those in a 2D culture. In a 2D culture, where cells adhere to the vessel surface and form a monolayer, it is not possible to accurately reflect the interactions between cells and the external environment as they would occur in a tumor mass in vivo. A 3D culture, in which spheroids are formed by multiple layers of cells, allows for a more accurate representation of the conditions prevailing in the body and a better understanding of the interactions between the tested drugs and cells, and therefore allows for a more accurate determination of sensitivity to a given substance than in the case of a 2D culture [22]. The cytotoxic effect obtained in the 2D study did not correspond to the viability results obtained in the 3D culture. In most cases, the calculated LC_50_ values for spheroids (S-LC_50_) were higher than the LC_90_ values calculated for the corresponding cell lines in 2D culture (Table 1) [1]. T24 spheroids were characterized by greater sensitivity to Mumio than SV-HUC-1 cells after all incubation times (Tab.1). In the case of the 5637 cell line, after 24 h incubation time, Mumio was more effective compared to control normal cells, which was expressed by selectivity index (Tab.1). After a 48 and 72-h incubation period, the SI for 5637 spheroids was close or below 1, indicating similar effectiveness against normal uroepithelial cells. The observed variations could be attributed to the stage of cancer advancement from which the cell lines originated (T24 – grade III; 5637 – grade II) [23]. Grade III cancers are characterized by rapid growth, extensive genetic alterations, and disrupted tissue organization, making them more susceptible to therapies that target cellular proliferation or genomic instability. In contrast, grade II cancers exhibit slower growth and retain more normal-like structural features, which can render them less responsive to treatments, especially in in vitro conditions [24]. To confirm this effect, more complex studies using organoids or animal models should be conducted.

There are few studies available that evaluate the effects and mechanisms of Mumio's action on cancer cells, and none of these studies have used 3D cell culture for testing. All analyses conducted to date have been based on standard 2D culture. Therefore, it is impossible to directly compare the results obtained in the described work with those presented in the studies.

Thawatchai et al. conducted a study in which they assessed how Mumio affects six different human cancer cell lines: breast (MDA-MB-231), lung (A549), cervix (HeLa), ovary (SKOV-3), liver (HepG2), and colon (SW-620) compared to the normal human lung fibroblast cell line MRC-5. They showed that Mumio had a stronger effect on almost all cancer cell lines, compared to the normal fibroblast line. The exception was the breast cancer cell line, for which the cytotoxicity of Mumio was comparable to the control group [25]. Barouji et al. assessed the effect of Mumio on two breast cancer cell lines (MCF-7 and MDA-MB-231) compared to normal epithelial cells (MCF-10A); thus, they compared the effect of Mumio between normal and cancer cells of the same organ. They found a concentration-dependent cytotoxic effect of Mumio on cancer cells, with a simultaneous lack of significant cytotoxicity towards normal cells. Moreover, the MDA-MB-231 line showed significant sensitivity to Mumio's action, which does not align with the results of Thawatchai et al. The difference between the results of the mentioned studies may be due to the use of different control lines [19]. In another study, doxorubicin-loaded Mumio-based nanocaries were tested on two breast cancer cell lines (MCF-7 and ZR-75–1). Such a formulation proved to be a promising carrier, inducing apoptosis in cancer cells and reducing the side effects of doxorubicin [26]. In another study, Mumio potentiated the effect of CMF (cyclophosphamide, methotrexate, 5-fluorouracil) treatment in the osteosarcoma rat model [27]. Tavassoli and Monsefi also confirmed the cytotoxic properties of Mumio towards cancer cells. They compared the effect of Mumio on the human cervical cancer cell line (HeLa) and normal human fibroblasts (NIH). The results obtained with the HeLa cell line were similar to those reported by Thawatchai et al. [28]. In most of the analyzed studies, Mumio was more effective against cancer than normal cells. In this study, we observed a similar effect; Mumio cytotoxicity was highest compared to ontrol for the T24 cell line after all incubation times, and for the 5637 cell line after 24 h incubation (Table 1). In the case of the 5637 cell line after 48 h and 72 h incubation with Mumio, Selectivity Index was comparable to control cells (Tab.1), however Ki67 expression, in those groups was reduced (Fig. 3C). This result may suggest that Mumio has a weaker effect on non-cancerous cells, however this effect should be more detailed analysed in future studies.

Despite the differences in gene expression between the tested cell lines, a specific mechanism of action of Mumio can be observed (Fig. 4A). Mumio leads to the inhibition of cell proliferation by downregulating topoisomerase II genes (TOP2A and TOP2B). An increase in CDKN1A (p21) and CDKN1B (p27) led to changes in the cell cycle, resulting in the arrest of G1/S and G2/M checkpoints. At this time, two defense mechanisms are activated simultaneously. On the one hand, the upregulation of topoisomerase III genes (TOP3A and TOP3B) and cell cycle kinases (CDK2 and CDK7) prepares cells for DNA repair and the continuation of the cell cycle. On the other hand, the upregulation of CASP9 and downregulation of BCL2 genes may indicate the induction of apoptosis. Downregulation of caspase 3 (CASP3), consistent with the luminescence assay result (Fig. 2D), and increased expression of caspase 9 may suggest the initiation of the apoptosis process without the effector phase. The proposed mechanism of action remains a hypothesis based on the analysis of a limited number of genes. In a previous study using the same cell lines cultured in 2D conditions, flow cytometry analysis revealed cell cycle inhibition at the G0/G1 and S phases in cancer cells [1]. Taken together, these findings suggest that Mumio may interfere with cell cycle progression. However, further comprehensive analyses are required to confirm the exact molecular mechanism by which Mumio regulates the cell cycle. Earlier studies conducted on other cancer cell lines in 2D culture have shown that Mumio induces apoptosis [1, 19, 20, 29, 30]. In the present study, performed using a 3D spheroid model, we analyzed the expression of five apoptosis-related genes (CASP3, CASP7, CASP9, BAX, and BCL2). Among them, only CASP9 was upregulated, and BCL2 was downregulated, while no statistically significant changes were observed in the remaining genes. These findings may suggest that, within the more complex architecture of spheroids, the Mumio concentration effective in 2D cultures is insufficient to trigger apoptosis. Further studies using higher Mumio concentrations or extended incubation times are necessary to evaluate this hypothesis. Molecular analysis also showed consistent downregulation of genes such as TOP2A and TOP2B across all three cell lines, suggesting a potentially important role in mediating the observed Mumio effect. Topoisomerase II is an essential enzyme that regulates DNA topology and is a common target in many anticancer therapies. Topoisomerase IIα plays a critical role in DNA replication, chromosome condensation, and segregation during mitosis, making it indispensable for the survival and proliferation of dividing cells. Topoisomerase IIβ, while sharing structural similarity with topoisomerase IIα, is more involved in transcriptional regulation and DNA repair in non-dividing cells. Targeting topoisomerase II can trigger further changes in gene expression, which, in consequence, can lead to cell cycle arrest and apoptosis [31]. To confirm this hypothesis, the expression of these genes should be restored, allowing determination of whether they are key to restoring normal cell function and confirming whether topoisomerase II is the actual target of Mumio.

Mumio demonstrates selective cytotoxicity toward bladder cancer cells in 2D culture, suggesting its potential in supportive therapy. This selectivity was confirmed in a 3D study, using a spheroid model, especially against the higher-grade T24 cell line. Mumio decreased cell viability and proliferation without significantly altering spheroid size or morphology. Two opposing molecular pathways appear to be activated simultaneously: one promoting cell survival and DNA repair, and the other driving the cells toward apoptosis. However, its lower cytotoxic effect (resulting from higher concentrations of Mumio necessary to reduce cell viability) in 3D culture, compared to standard 2D culture, indicates that its efficacy may be limited in complex tumor environments. These findings highlight the need for further investigation into Mumio’s bioavailability, penetration capacity, and mechanism of action within three-dimensional tumor structures before considering clinical application.

## Supplementary Information

Additional file 1: Supplementary Table 1. Fold changes (FC) of analyzed genes after incubation of SV-HUC-1 spheroids with different Mumio concentrations. With green indicating strong, orange moderate, and blue weak changes in expression of the tested genes. Data are from four biological replicates (*n* = 4).

Additional file 2: Supplementary Table 2. Fold changes (FC) of analyzed genes after incubation of T24 spheroids with different Mumio concentrations. With green indicating strong, orange moderate, and blue weak changes in expression of the tested genes. Data are from four biological replicates (*n* = 4).

Additional file 3: Supplementary Table 3. Fold changes (FC) of analyzed genes after incubation of 5637 spheroids with different Mumio concentrations. With green indicating strong, orange moderate, and blue weak changes in expression of the tested genes. Data are from four biological replicates (*n* = 4).

Additional file 4: Supp.Fig.1. Schematic representation of RT-PCR plate. A plate layout of tested genes, together with reference genes, was presented. 

Additional file 5: Supp.Fig.2. Cytotoxic effect of Mumio on tested spheroids. A – Spheroids morphology, including all tested Lethal Concentration for T24, 5637, and SV-HUC-1 cell lines. B – Slight changes in the spheroids' surface after incubation with Mumio were observed. C – A lack of dependency was observed between incubation time and spheroid viability for the tested cell lines. The values are presented as means ± SD. *p<0.05, **p<0.01, ***p<0.001,****p<0.0001. Data are from four biological replicates (*n* = 4).

Additional file 5: Supp.Fig.3. Histological and immunohistochemical evaluation. Haemoxylin and eosin, together with Ki67 staining, for all tested Lethal Concentrations and incubation times. Data are from four biological replicates (*n* = 3).

## Data Availability

All data generated or analysed during this study are included in this published article and its supplementary information files.

## Abbreviations

cDNA: Complementary DNA
DNA: Deoxyribonucleic acid
FDA: Food and Drug Administration
HE: Hematoxylin-eosin
LC: Lethal Concentration
PCR: Polymerase Chain Reaction
qPCR: Quantitative PCR
RNA: Ribonucleic acid
S-LC: Spheroid Lethal Concentration
SI: Selectivity Index
WHO: World Health Organization
