# Supplementary material for: Mumio and bladder cancer: unlocking its potential in 3D cell culture
Source: BMC Cancer. 2026 Mar 14;26:510. doi: 10.1186/s12885-026-15838-1 (PMC13101165; doi:10.1186/s12885-026-15838-1)
Supplement: Supplementary file 1 — Additional file 1: Supplementary Table 1. Fold changes (FC) of analyzed genes after incubation of SV-HUC-1 spheroids with different Mumio concentrations. With green indicating strong, orange moderate, and blue weak changes in expression of the tested genes. Data are from four biological replicates (n = 4). [file 12885_2026_15838_MOESM1_ESM.docx]

**Supplementary Table 1.** Fold changes (FC) of analyzed genes after incubation of SV-HUC-1 spheroids with different Mumio concentrations. With green indicating strong, orange moderate, and blue weak changes in expression of the tested genes. Data are from four biological replicates (n = 4).

| **SV-HUC-1** | | | | |
| --- | --- | --- | --- | --- |
| *Gene* | *LC* | *FC (Mean)* | *SD* | *p-value* |
| TOP1 | 50 | 0.6475 | 0.1726 | 0.0027 |
| TOP2A | 10 | 0.8252 | 0.05938 | 0.0429 |
|  | 50 | 0.6217 | 0.1177 | 0.0001 |
|  | 90 | 0.5722 | 0.09365 | <0,0001 |
| TOP2B | 50 | 0.6088 | 0.08926 | <0,0001 |
|  | 90 | 0.6529 | 0.09005 | 0.0002 |
| TOP3A | 90 | 1.407 | 0.1595 | 0.0037 |
| TOP3B | 90 | 1.557 | 0.09156 | <0,0001 |
| CASP3 | 50 | 0.672 | 0.1769 | 0.0046 |
|  | 90 | 0.7547 | 0.07 | 0.0443 |
| CASP9 | 90 | 1.561 | 0.3205 | 0.0067 |
| CDK2 | 90 | 1.218 | 0.05573 | 0.008 |
| CDK7 | 90 | 1.273 | 0.0388 | <0,0001 |
| CDKN1A | 90 | 1.371 | 0.1815 | 0.0106 |

LC – Lethal Concentration; SD – Standard Deviation.
