# Supplementary material for: Mumio and bladder cancer: unlocking its potential in 3D cell culture
Source: BMC Cancer. 2026 Mar 14;26:510. doi: 10.1186/s12885-026-15838-1 (PMC13101165; doi:10.1186/s12885-026-15838-1)
Supplement: Supplementary file 2 — Additional file 2: Supplementary Table 2. Fold changes (FC) of analyzed genes after incubation of T24 spheroids with different Mumio concentrations. With green indicating strong, orange moderate, and blue weak changes in expression of the tested genes. Data are from four biological replicates (n = 4). [file 12885_2026_15838_MOESM2_ESM.docx]

**Supplementary Table 2.** Fold changes (FC) of analyzed genes after incubation of T24 spheroids with different Mumio concentrations. With green indicating strong, orange moderate, and blue weak changes in expression of the tested genes. Data are from four biological replicates (n = 4).

| **T24** | | | | |
| --- | --- | --- | --- | --- |
| *Gene* | *LC* | *FC (Mean)* | *SD* | *p-value* |
| TOP1 | 90 | 1.433 | 0.263 | 0.0083 |
| TOP2A | 10 | 0.58 | 0.02217 | <0,0001 |
|  | 50 | 0.278 | 0.02232 | <0,0001 |
|  | 90 | 0.1209 | 0.01557 | <0,0001 |
| TOP3B | 90 | 1.346 | 0.1425 | 0.0165 |
| CASP9 | 90 | 1.545 | 0.2164 | 0.0013 |
| BCL2 | 50 | 0.6453 | 0.1857 | 0.0301 |
| CDK1 | 10 | 0.6316 | 0.01971 | <0,0001 |
|  | 50 | 0.3205 | 0.01836 | <0,0001 |
|  | 90 | 0.1251 | 0.02493 | <0,0001 |
| CDK2 | 50 | 0.6365 | 0.2095 | 0.0183 |
| CDK4 | 50 | 0.6453 | 0.1857 | 0.0301 |
| CDK7 | 90 | 1.723 | 0.4145 | 0.0032 |
| CDKN1A | 90 | 4.239 | 2.059 | 0.0039 |
| CDKN1B | 90 | 1.426 | 0.3015 | 0.0444 |

LC – Lethal Concentration; SD – Standard Deviation.
