# Supplementary figures and images for: Mumio and bladder cancer: unlocking its potential in 3D cell culture
Source: BMC Cancer. 2026 Mar 14;26:510. doi: 10.1186/s12885-026-15838-1 (PMC13101165; doi:10.1186/s12885-026-15838-1)

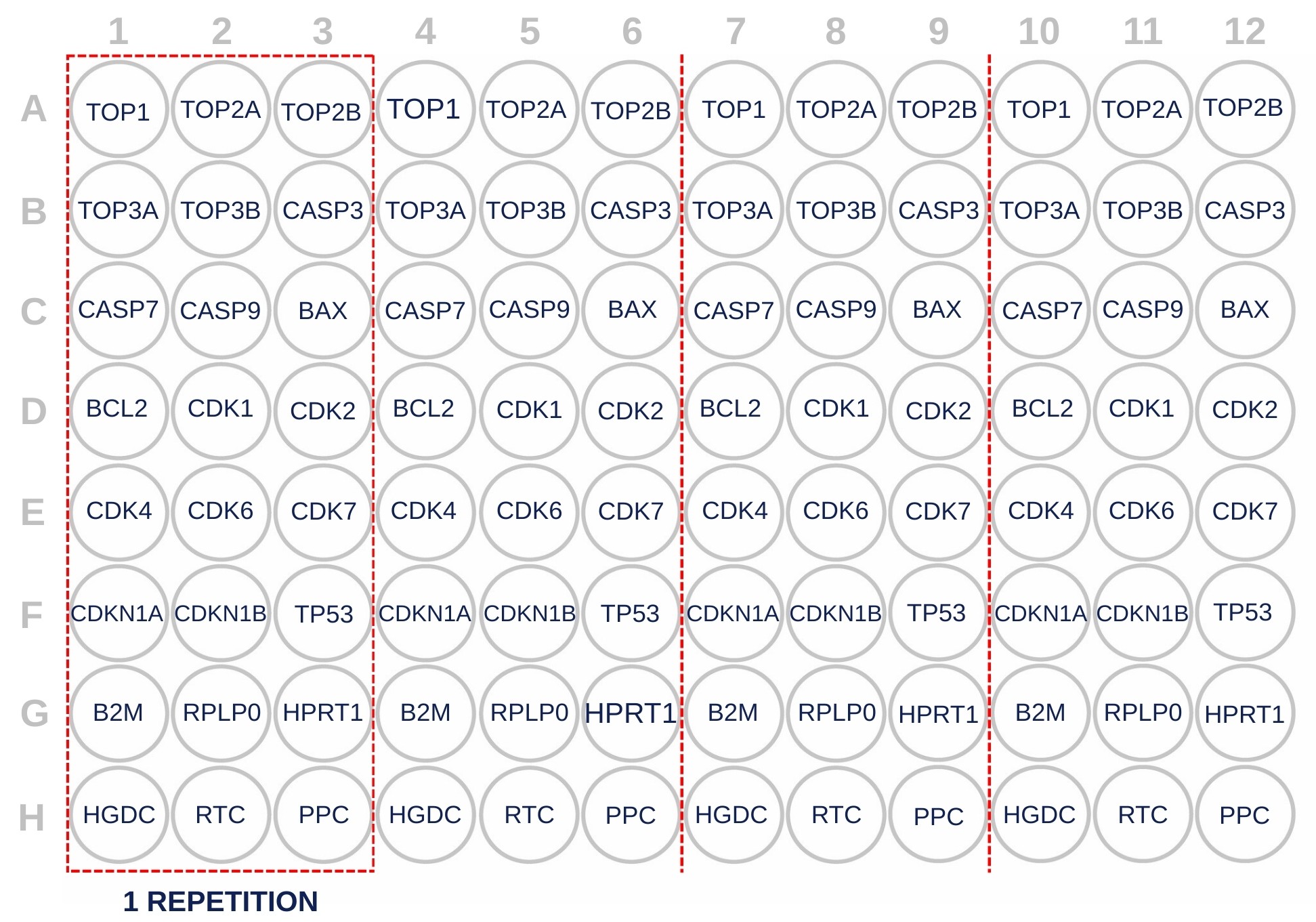

Supplement: Supplementary file 4 — Additional file 4: Supp.Fig.1. Schematic representation of RT-PCR plate. A plate layout of tested genes, together with reference genes, was presented. [file 12885_2026_15838_MOESM4_ESM.jpg]

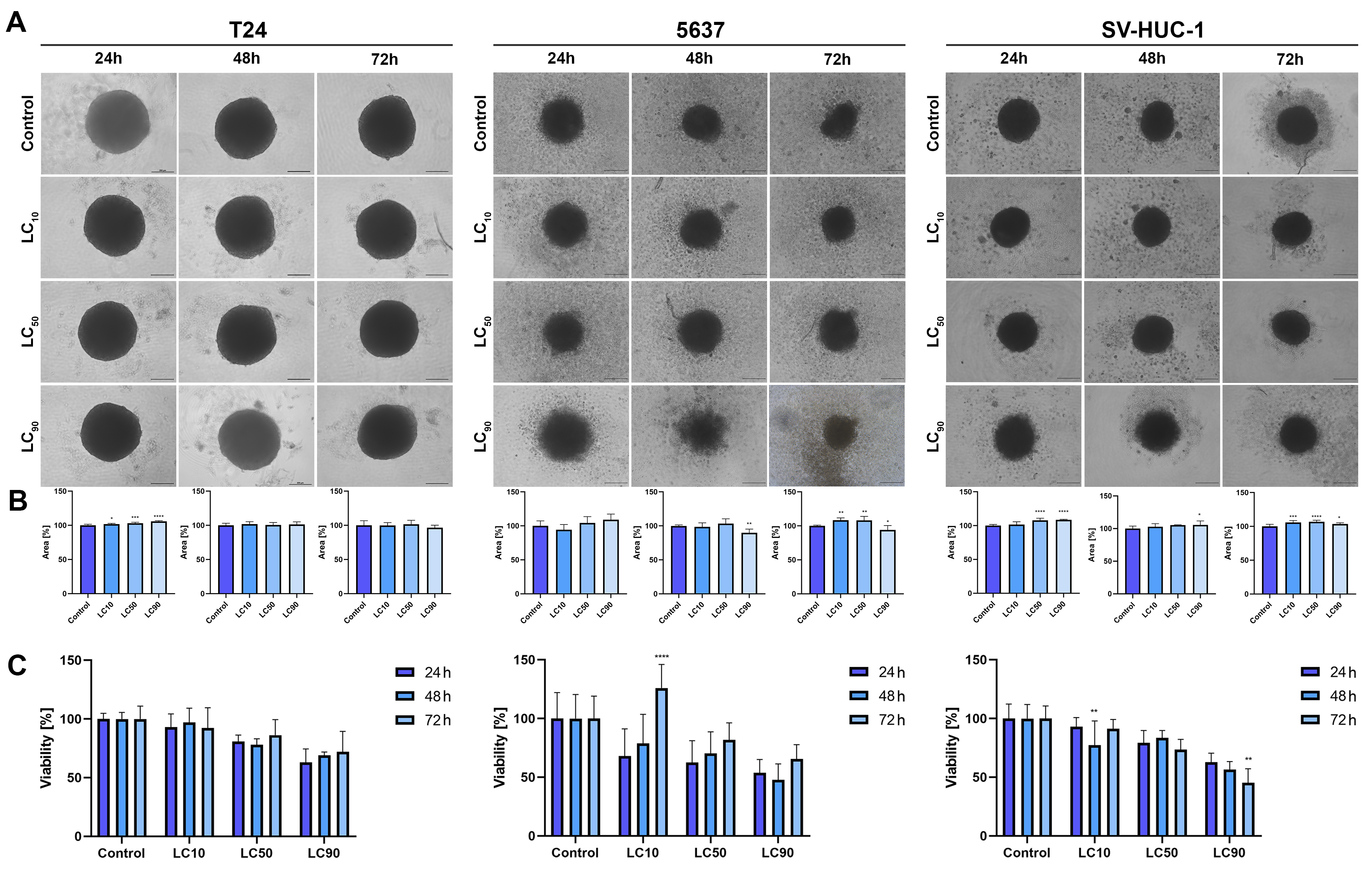

Supplement: Supplementary file 5 — Additional file 5: Supp.Fig.2. Cytotoxic effect of Mumio on tested spheroids. A – Spheroids morphology, including all tested Lethal Concentration for T24, 5637, and SV-HUC-1 cell lines. B – Slight changes in the spheroids' surface after incubation with Mumio were observed. C – A lack of dependency was observed between incubation time and spheroid viability for the tested cell lines. The values are presented as means ± SD. *p<0.05, **p<0.01, ***p<0.001,****p<0.0001. Data are from four biological replicates (n = 4). [file 12885_2026_15838_MOESM5_ESM.tif]

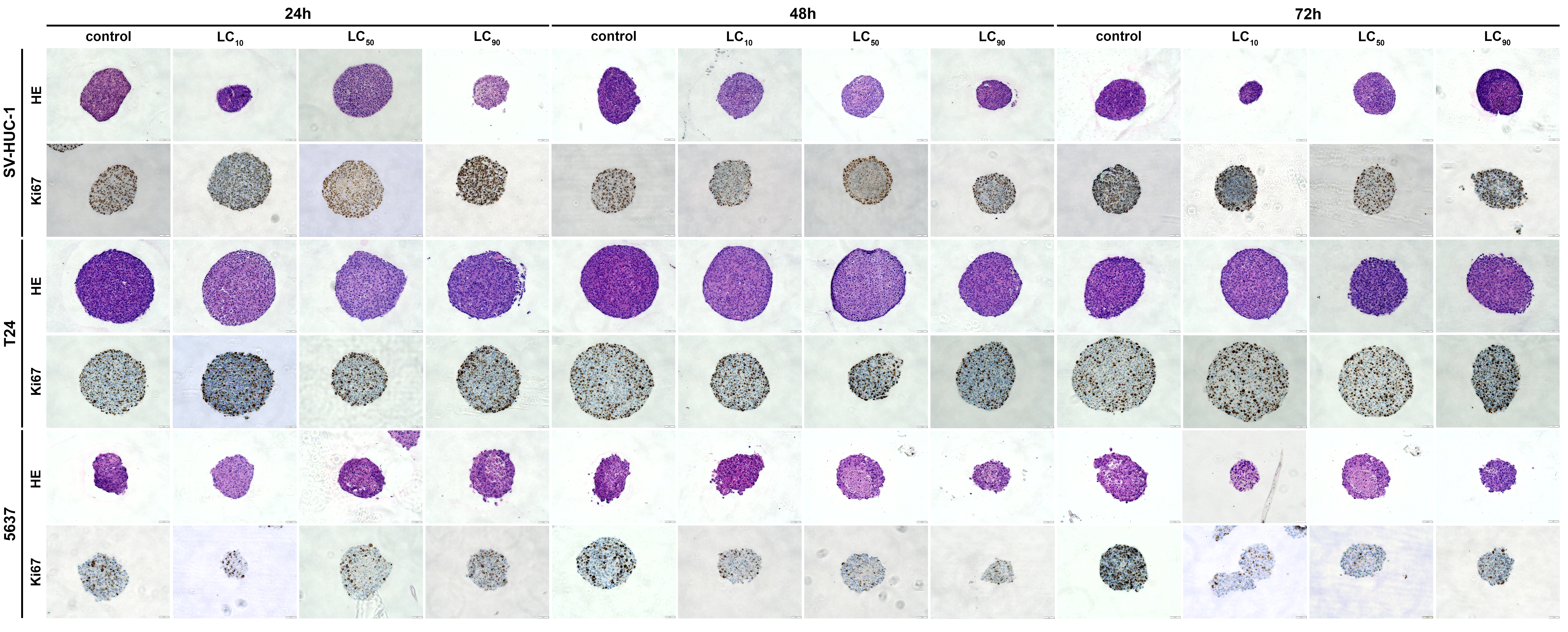

Supplement: Supplementary file 6 — Additional file 5: Supp.Fig.3. Histological and immunohistochemical evaluation. Haemoxylin and eosin, together with Ki67 staining, for all tested Lethal Concentrations and incubation times. Data are from four biological replicates (n = 3). [file 12885_2026_15838_MOESM6_ESM.tif]
